# Analysis of Dietary Patterns Containing Curcumin and Oral Cancer

**DOI:** 10.3290/j.ohpd.c_2832

**Published:** 2026-09-29

**Authors:** Yi-Yang Zhao, Yuan Lai, Hao-Ran Duan, Zi-Ying Ji, Tian-Ke Li, Wei Sun

**Affiliations:** a Yi-Yang Zhao Dentist, Department of Stomatology,The Second Hospital of Shijiazhuang, Shijiazhuang, Hebei, P.R. China. Conceptualization.; b Yuan Lai Dentist, Department of Stomatology, The Fourth Affiliated Hospital of Hebei Medical University, Shijiazhuang, Hebei, P.R. China. Data curation.; c Zi-Ying Ji Dentist, Department of Stomatology, Baoding Fourth Central Hospital, Baoding, Hebei, P.R. China. Methodology.; d Hao-Ran Duan Dentist, Department of Stomatology, The Fourth Affiliated Hospital of Hebei Medical University, Shijiazhuang, Hebei, P.R. China. Software.; e Tian-Ke Li Dentist, Department of Stomatology, The Fourth Affiliated Hospital of Hebei Medical University, Shijiazhuang, Hebei, P.R. China. Wrote original draft.; f Wei Sun Dentist, Department of Stomatology, The Fourth Affiliated Hospital of Hebei Medical University, Shijiazhuang, Hebei, P.R. China. Reviewed and edited the manuscript.

**Keywords:** curcumin, logistic regression, oral cancer, risk factors, ROC curve trend test, SHAP-value.

## Abstract

**Purpose:**

This study aims to: (1) quantify the association between dietary intake of curcumin-containing foods (oranges and carrots) and the risk of oral cancer using NHANES data; (2) identify the consumption frequency threshold associated with reduced oral cancer risk; and (3) evaluate the predictive performance of a model incorporating curcumin-related dietary factors.

**Materials and Methods:**

This study is based on the 2003-2004 cycle data of the National Health and Nutrition Examination Survey (NHANES) in the United States, and a total of 2762 research subjects were included. Multiple statistical methods were comprehensively employed, including descriptive statistics to present differences in demographic characteristics and lifestyles, correlation analysis to measure the degree of association between variables, logistic regression analysis to identify the independent association between dietary intake of curcumin and the risk of oral cancer, trend test to determine whether there is a significant linear trend between dietary intake of curcumin and the risk of oral cancer, 3D bubble chart to visually display the interaction effect of the frequency of intake of two types of foods on the risk of oral cancer, SHAP-value analysis to quantify the influence of features on model output, confusion matrix and ROC curve to evaluate model performance, and a comprehensive analysis of the role of dietary intake of curcumin-containing foods in the onset of oral cancer.

**Results:**

The results show that there are significant differences between the oral cancer group and the non-oral cancer group in terms of social economic status, lifestyle, and dietary intake of curcumin-containing foods. Social economic disadvantage may be associated with an increased risk of oral cancer, and the proportion of not consuming curcumin-containing foods is higher in the oral cancer group. Correlation analysis indicates that education level, poverty-income ratio, and dietary intake of foods containing curcumin such as oranges and carrots are negatively correlated with oral cancer, while smoking and drinking have positive correlations. Logistic regression analysis reveals that occasional consumption of oranges, frequent consumption of oranges, etc., rich in curcumin, are associated with the risk of oral cancer. Trend test analysis shows that as the frequency of intake of oranges and carrots increases, the proportion of oral cancer cases decreases (all p-values are all less than 0.01), indicating a statistically significant negative correlation between the frequency of intake and the prevalence of oral cancer. Interaction analysis shows that high-frequency intake of oranges or carrots is associated with a lower risk of oral cancer, and high-frequency intake of both is associated with a lower risk, while low-frequency intake of both is associated with a higher risk. SHAP-value analysis highlights the key role of dietary intake of curcumin-containing foods in the model, with the weighted precision rate, recall rate, and F1 score of the model all above 0.96, indicating strong predictive ability for both sample types. The ROC curve verifies that the model AUC reaches 0.89, demonstrating good predictive performance and the ability to effectively identify high-risk individuals for oral cancer.

**Conclusion:**

Dietary conditions containing curcumin are closely related to the risk of oral cancer and can serve as a potential predictive indicator. For clinical practice, we suggest that regular consumption of curcumin-containing foods (oranges or carrots ≥3 times per week, corresponding to an estimated daily curcumin intake of 20–40 mg) may be beneficial for oral cancer prevention. For future research, prospective cohort studies with quantitative dietary assessment and pharmacokinetic studies on curcumin bioavailability are needed to validate these findings.

Oral cancer, as one of the common malignant tumors worldwide, has a significant impact on the quality of life and survival rate of patients. According to statistics from the World Health Organization, approximately several hundred thousand new cases of oral cancer are diagnosed each year, and its incidence rate is on the rise in some regions.^5^ In Europe, oral cancer is also one of the major types of cancer, accounting for a certain proportion of all cancer cases. In the United States, oral cancer is also one of the leading causes of hospitalization and death, causing significant economic losses every year.^9,17^ In recent years, with the changes in lifestyle and the aging of the population, the incidence of oral cancer has shown a significant upward trend in Asian countries, especially in populous countries such as China and India.^14^

Traditional risk factors such as smoking and alcohol consumption play a significant role in the onset of oral cancer. Smoking, as a definite carcinogenic factor, can cause damage to the oral mucosa and promote canceration, significantly increasing the incidence of oral cancer.^10^ Drinking alcohol can also irritate the oral mucosa. Prolonged and excessive alcohol consumption will increase the risk of developing oral cancer.^22^ Furthermore, family history is also an important factor in the occurrence of oral cancer. People with a family history of cancer tend to have a higher genetic susceptibility.^5^

Recent advances have highlighted the role of biological biomarkers, oral microbiome dysbiosis, and host responses in oral carcinogenesis. For instance, Radaic et al^19^ comprehensively reviewed biological biomarkers of oral cancer, emphasizing the potential of non-invasive diagnostic approaches. Belibasakis et al^1^ demonstrated bacteriome and mycobiome dysbiosis in oral mucosal dysplasia and oral cancer. Furthermore, Soghli et al^24^ discussed recent advancements in artificial intelligence-powered cancer prediction from oral microbiome, offering new perspectives for risk stratification. Additionally, the relationship between oral health and systemic diseases has been increasingly recognized. Isola et al^11^ conducted a bibliometric analysis of the association between periodontitis and systemic diseases. Eke et al^7,8^ developed a scoring algorithm for predicting periodontitis using NHANES self-report measures and also examined the association between missing teeth and all-cause mortality in US adults. Peng and Peng investigated the association between stress hyperglycemia ratio and periodontitis among diabetic patients using NHANES data.^18^ Jia and Chen explored the relationship between the Composite Dietary Antioxidant Index and periodontitis.^12^ These findings underscore the importance of integrating multiple biological dimensions, including dietary factors, into oral cancer prediction models.

Apart from traditional factors, dietary factors also play a significant role in the occurrence of oral cancer. In recent years, more and more studies have begun to focus on the potential anti-cancer effects of curcumin. Curcumin is a natural compound with antioxidant, anti-inflammatory and anti-cancer properties, and is widely present in foods such as turmeric, oranges, and carrots. For example, studies have found that curcumin can inhibit the proliferation and invasion of oral cancer cells and induce cell apoptosis.^21^ Research has confirmed that curcumin has a certain preventive effect on oral cancer.^17^ Studies have shown that curcumin inhibits the growth of oral cancer cells by down-regulating the PI3K/AKT/mTOR signaling pathway.^3^ The research has found that curcumin has an anti-cancer effect on oral squamous cell carcinoma.^15^ The study found that curcumin induces apoptosis and inhibits proliferation of oral cancer cells through the mitochondrial pathway.^9^

We hypothesized that higher consumption frequency of curcumin-containing foods is associated with lower odds of oral cancer. Therefore, the specific aims of this study were: (1) to compare the baseline characteristics and dietary patterns between oral cancer and non-oral cancer groups; (2) to assess the dose-response relationship between curcumin-containing food intake frequency and oral cancer risk using trend tests; (3) to evaluate the predictive performance of a random forest model incorporating curcumin-related dietary factors; and (4) to estimate a potential preventive intake threshold for future clinical and public health recommendations.

## MATERIALS AND METHODS 

Approval was obtained from the the Institutional Review Board of The Fourth Hospital of Hebei Medical University, Ethics Number: 2025KT027. The procedures used in this study adhere to the tenets of the Declaration of Helsinki.

### Clinical Data 

The data for this study were sourced from the National Health and Nutrition Examination Survey (NHANES) of the United States (https://www.cdc.gov/nchs/nhanes/index.htm). This was a cross-sectional survey conducted by the National Center for Health Statistics (NCHS) and the Centers for Disease Control and Prevention. The aim of NHANES was to provide nationally representative data on the civilian population of the United States. The Ethics Review Committee of NCHS approved the data collection protocol and all survey participants gave informed consent before undergoing interviews and examinations. In this study, a period (2003-2004) of NHANES responses’ public data files were used to construct a dataset. The study population included all NHANES respondents.

### Data Collection 

This study aims to explore the disease of oral cancer, while considering a series of potential covariates. These variables are based on previous literature and substantive reasoning and are believed to have potential associations with the disease of oral cancer. The study adopted a standardized questionnaire to collect information on various factors including age, gender, race, marital status, smoking status, drinking status, and the ratio of family poverty income. For dietary assessment, the frequency of consuming curcumin-containing foods (oranges and carrots) was categorized based on the NHANES Dietary Questionnaire data. The response options were defined as follows: “Never eat” (0 times per week), “Rarely eat” (1–2 times per month), “Occasionally eat” (1–2 times per week), and “Frequently eat” (≥3 times per week). These definitions were applied consistently for both orange and carrot consumption variables. For the missing values in the data, we used interpolation methods through R software for supplementation.Whether one has the disease of oral cancer is assessed through a series of self-reported questionnaires. Based on affirmative answers to the multiple-choice questions (MCQ240Q) of NHANES: “At what age was the first diagnosis of oral cancer, tongue cancer or lip cancer made?” Through comprehensive analysis of these data, this study aims to analyze the relationship between the dietary situation of foods containing curcumin and the disease of oral cancer.

### Statistical Analysis 

All statistical analyses were conducted in accordance with the guidelines of the CDC (Centers for Disease Control and Prevention) and used sampling weights to generate national representative prevalence estimates for the uninstitutionalized US population. Continuous variables were presented in the form of mean ± standard deviation (SD), while for categorical variables, the results were presented as numbers and percentages; for normally distributed quantitative data, the mean ± SD were used for description, and the T-test was used to evaluate differences between groups; for non-normally distributed data, the median and interquartile range were used for description. Weighted t-tests (for continuous variables) or weighted chi-squared tests (for categorical variables) were used to compare differences between the two groups. A logistic regression model was constructed to calculate odds ratios and confidence intervals to assess risks; SHAP (SHapley Additive exPlanations) values were used to quantify the impact of features on model outputs; a random forest model was constructed to determine important feature indicators, and an ROC curve analysis was conducted to verify that the random forest model had good predictive performance for oral cancer, fully ensuring the robustness of the research conclusions.

## RESULTS

The datasets generated during and/or analyzed during the current study are available from the corresponding author on reasonable request.

### Baseline Characteristics 

This study included a total of 2762 research subjects, among which there were 59 cases in the oral cancer group and 2703 cases in the non-oral cancer group. The baseline characteristics showed no statistically significant differences in terms of demographics, such as age, gender, race, and marital status (p > 0.05). However, there were statistically significant differences in other demographic factors or diets containing curcumin and lifestyle (p < 0.001). The specific distribution of characteristics is as follows: in terms of social economic status, the proportion of those with a 9^th^- to 11^th^-grade education in the oral cancer group was 32.2% (13.2% in the non-oral cancer group), and the poverty ratio was 1.22 (2.42 in the non-oral cancer group), a statistically significant difference, reflecting that social economic disadvantage may be associated with the oral cancer. In the oral cancer group, the proportion of participants who never consumed curcumin-containing foods was higher than that in the non-oral cancer group, and the difference was statistically significant (p < 0.001), suggesting that consuming foods containing curcumin may improve the qualilty of life of oral cancer patients. The proportion of those who currently smoked in the oral cancer group was the highest, again confirming the oral toxicity effect of tobacco exposure; the proportion of those who occasionally drank alcohol in the oral cancer group was the highest, suggesting that there is a certain association between drinking habits and the risk of oral cancer (Table 1).

**Table 1:** Baseline characteristics

Variable		Oral cancer		p-value
		No (n=2703)	Yes (n=59)	
**Age**		51 (35~68)	46 (37~61)	0.205
**Gender**	Male	1251 (46.3%)	21 (35.6%)	0.103
	Female	1452 (53.7%)	38 (64.4%)	
**Race**	Non-Hispanic White	1544 (57.1%)	27 (45.8%)	0.364
	Non-Hispanic Black	482 (17.8%)	15 (25.4%)	
	Mexican American	528 (19.5%)	13 (22.0%)	
	Other races	81 (3.0%)	3 (5.1%)	
	Other Hispanics	68 (2.5%)	1 (1.7%)	
**Educational level**	< high school	339 (12.5%)	13 (22.0%)	<0.001
	Nine to eleven years	357 (13.2%)	19 (32.2%)	
	High school	663 (24.5%)	11 (18.6%)	
	College or above	1344 (49.8%)	16 (27.1%)	
**Marital status**	Single	376 (13.9%)	10 (16.9%)	0.056
	Married	1565 (57.9%)	24 (40.7%)	
	Divorced	248 (9.2%)	11 (18.6%)	
	Widowed	286 (10.6%)	6 (10.2%)	
	Separated	66 (2.4%)	2 (3.4%)	
	Cohabiting	162 (6.0%)	6 (10.2%)	
**Income:poverty ratio**		2.42 (1.29~4.26)	1.22 (0.95~1.83)	<0.001
**Orange consumption**	Never	335 (12.4%)	28 (47.5%)	<0.001
	Rarely	1376 (50.9%)	17 (28.8%)	
	Occasionally	743 (27.5%)	11 (18.6%)	
	Frequently	249 (9.2%)	3 (5.1%)	
**Carrot consumption**	Never	248 (9.2%)	23 (39.0%)	<0.001
	Rarely	996 (36.8%)	21 (35.6%)	
	Occasionally	1114 (41.2%)	13 (22.0%)	
	Frequently	345 (12.8%)	2 (3.4%)	
**Smoke**	Never	1389 (51.4%)	22 (37.3%)	<0.001
	Former smoker	778 (28.8%)	8 (13.6%)	
	Current moker	536 (19.8%)	28 (47.5%)	
**Alcohol consumption**	Never	869 (32.1%)	12 (20.3%)	<0.001
	Occasionally	1339 (49.5%)	22 (37.3%)	
	Regularly	152 (5.6%)	5 (8.5%)	
	Heavy	343 (12.7%)	8 (13.6%)	


### Correlation Between Multiple Factors and Oral Cancer 

Based on the correlation analysis of oral cancer and various factors, we found that educational level was statistically significantly negatively correlated with the occurrence of oral cancer (r = -0.081, p<0.001). The poverty-income ratio was also statistically significantly negatively correlated with the occurrence of oral cancer (r = -0.080, p<0.001). A diet of consuming foods containing curcumin such as oranges and carrots was statistically significantly negatively correlated with oral cancer (p<0.001). Smoking status was statistically significantly positively correlated with oral cancer (r = 0.071, p<0.001), which means that the risk of oral cancer for smokers is higher than that for non-smokers. Alcohol consumption status was statistically significantly positively correlated with oral cancer (r = 0.080, p<0.001), which means that the risk of oral cancer for drinkers is higher than that for non-drinkers (Table 2).

**Table 2:** Correlation coefficients of multiple factors with oral cancer

Variable	Correlation coefficient	p-value
Educational level	-0.081**	<0.001
Income:poverty ratio	-0.111**	<0.001
Orange consumption	-0.92**	<0.001
Carrot consumption	-0.110**	<0.001
Smoke	0.071**	<0.001
Alcohol consumption	0.080**	<0.001
**When the confidence level (double test) is 0.001, the correlation is statistically significant.		

### Binary Logistic Regression Analysis of Two Curcumin-containing Diets

Binary logistic regression analysis was used to explore the association between dietary intake of foods containing curcumin and oral cancer. The logistic regression model calculated the odds ratio (OR) and its 95% confidence interval (CI) to screen out the factors that might be related to the risk of oral cancer. After adjusting for potential confounding factors, compared with participants who never consumed curcumin-containing foods (reference group), participants in the other consumption categories showed lower odds of oral cancer (p<0.05; Table 3). This indicates that as the intake of curcumin increased, the incidence of oral cancer decreased.

**Table 3:** Logistic regression analysis of factors associated with oral cancer in diets containing curcumin

Variable		OR (95%CI)	p-value
Orange consumption	Rarely	0.188 (0.100~0.354)	<0.001
	Occasionally	0.277 (0.132~0.581)	<0.001
	Frequently	0.243 (0.071~0.830)	0.024
Carrot consumption	Rarely	0.307 (0.164~0.574)	<0.001
	Occasionally	0.183 (0.088~0.379)	<0.001
	Frequently	0.083 (0.019~0.364)	<0.001


### Impact of Two Diets on Oral Cancer

As shown in Fig 1, the proportion of oral cancer cases statistically significantly decreased with increasing consumption frequency of both oranges and carrots (p for trend < 0.01 for both), i.e., negative dose-response relationship was observed.

**Fig 1:**
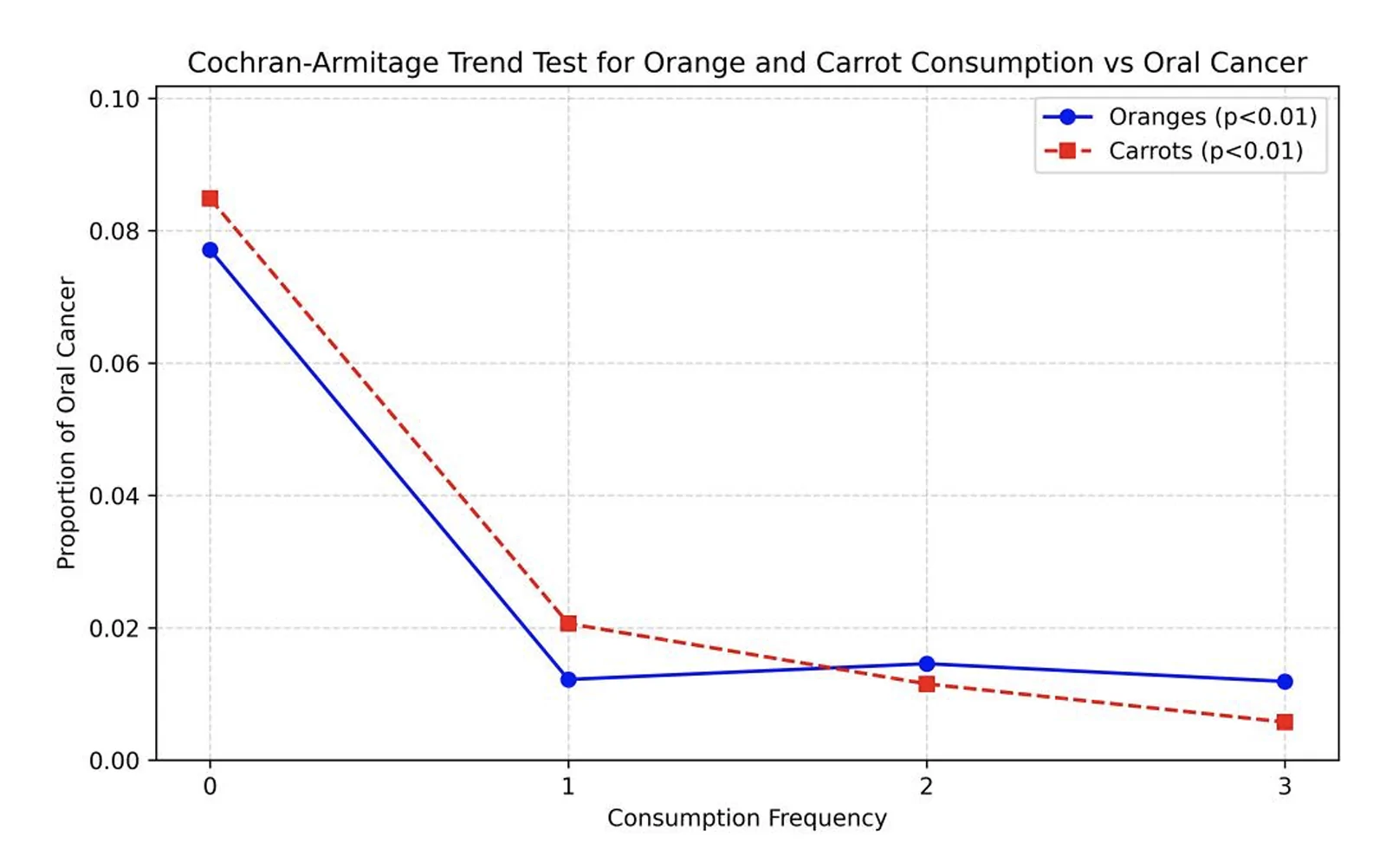
Cochran-Armitage Trend Test for the effect of orange and carrot consumption on oral cancer. The solid blue line is for oranges (p<0.01) and the dashed red line for carrots (p<0.01). The results indicate a statistically significant decrease in the proportion of oral cancer with increasing consumption frequency of both oranges and carrots.

### Interaction Between the Two Diets

Figure 2 visually demonstrates the interaction between orange and carrot intake. High-frequency consumption of either food was associated with lower oral cancer risk, while low-frequency consumption of both was associated with higher risk.

**Fig 2:**
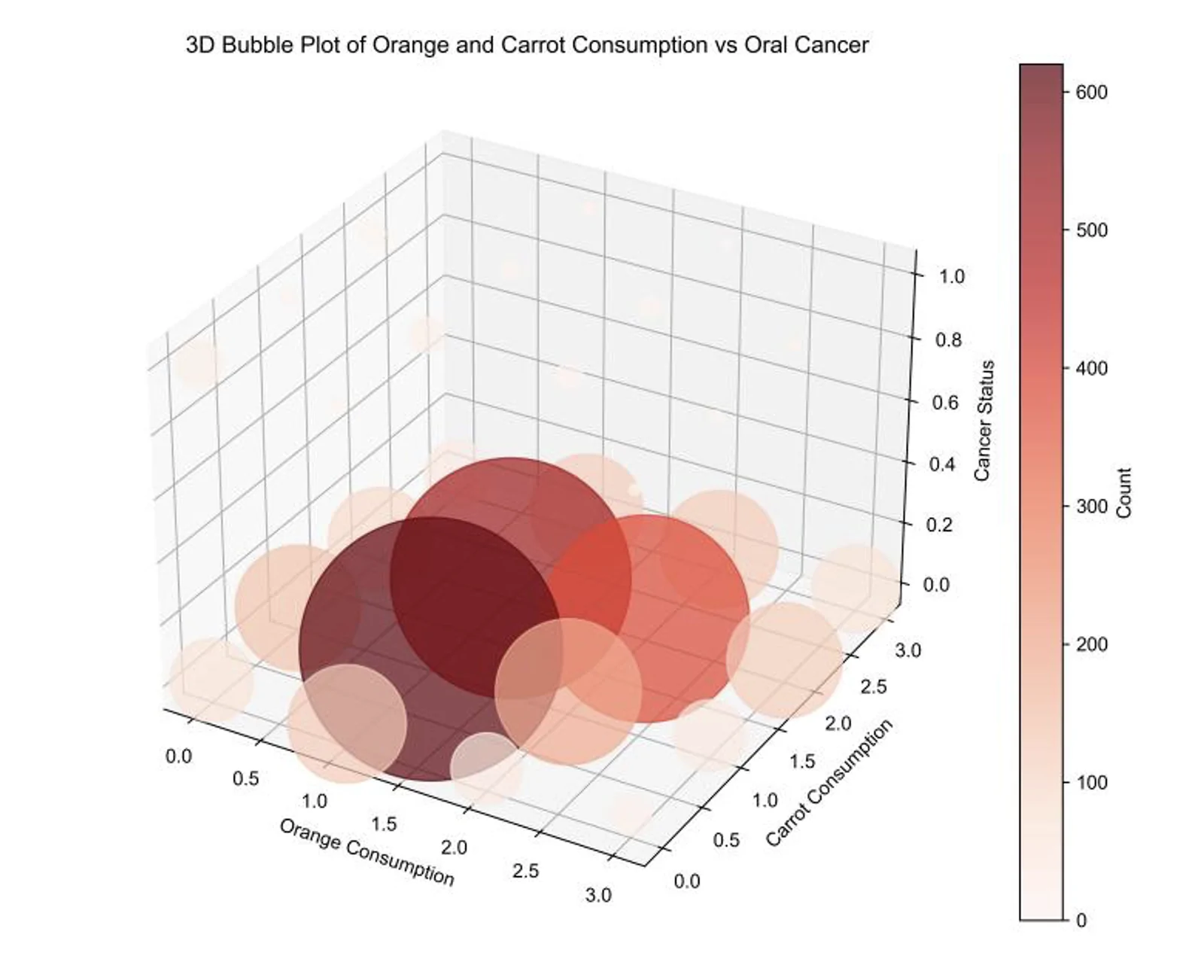
3D bubble plot illustrating the relationship between orange and carrot consumption and oral cancer. The x-axis represents orange consumption with values from 0 to 3, and the y-axis represents carrot consumption with values from 0 to 3. The z-axis represents cancer status through bubble size and color. The color bar on the right indicates the number of samples, with colors ranging from light to dark and numbers from low to high. Bubbles that are darker and larger indicate more samples. In the plot, larger and darker bubbles are seen in regions with low consumption of both oranges and carrots for cancer cases, while in regions with high consumption of both, larger and darker bubbles represent non-cancer cases. This suggests that high-frequency intake of oranges and carrots is associated with a lower risk of oral cancer.

### Distribution of Multi-feature SHAP Values

The SHAP summary plot in Fig 3 shows the impact of each feature on model output. For curcumin-containing foods (oranges and carrots), most samples had low SHAP-values, but red points (high feature values) appeared when intake frequency was high, indicating a notable impact on predictions.

**Fig 3:**
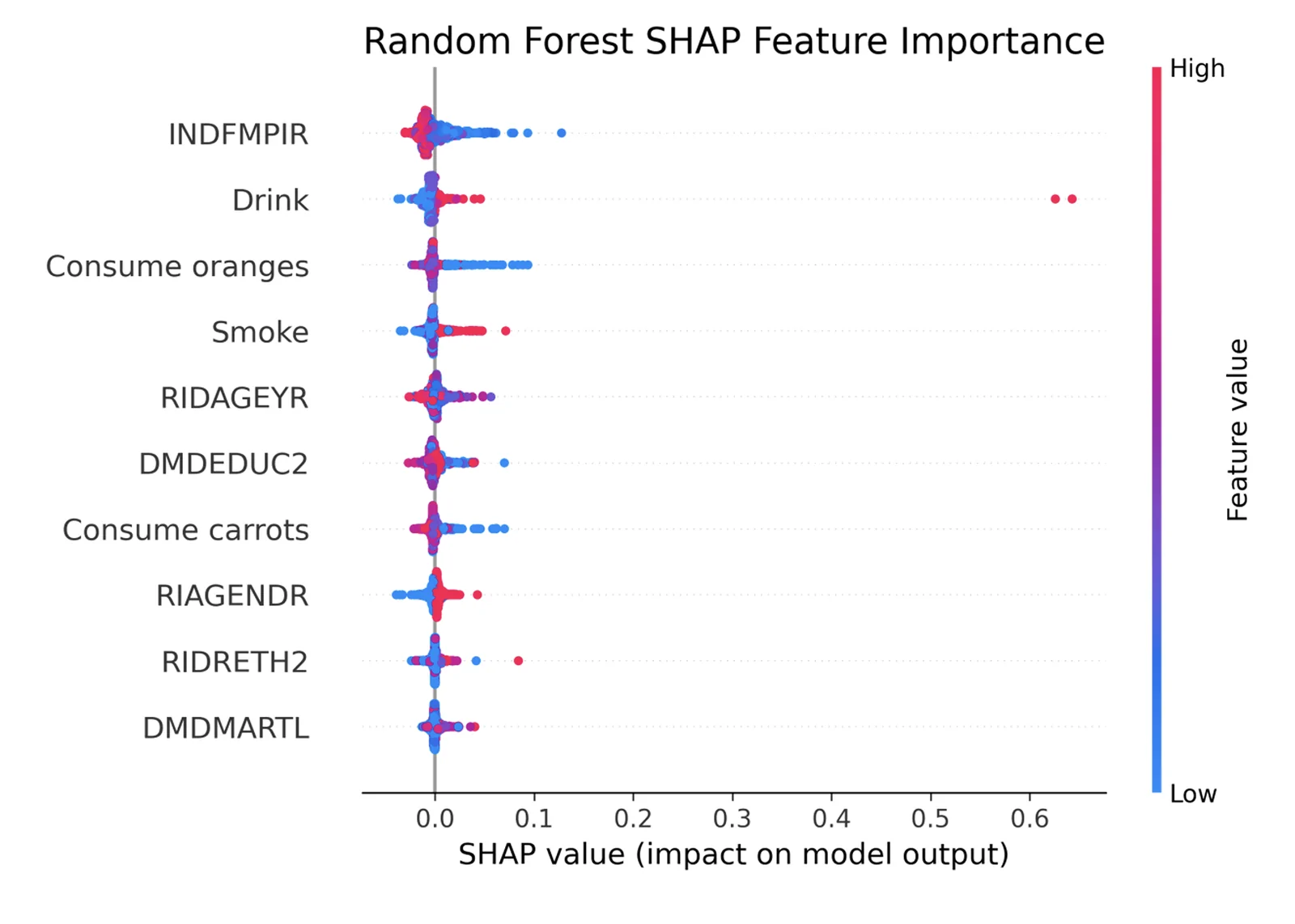
Distribution of SHAP-values for each feature, visualizing the impact of different features on the predictive model’s output for oral cancer. Each point represents a feature’s contribution to the model’s prediction for an individual sample. The x-axis indicates the SHAP-value, reflecting the impact of each feature on the model’s output, while the y-axis lists the features. The color gradient from blue to red indicates the feature value from low to high. The plot shows how each feature contributes to the prediction, either increasing or decreasing the predicted risk of oral cancer. Features with higher SHAP-values have a greater influence on the model’s predictions.

### Key Feature Value Diagrams of Each Characteristic During the Model Standardization Process

As shown in Fig 4, consumption of curcumin-containing foods was a key factor influencing model output, with importance values of 0.013 and 0.012, ranking second only to education level among all features.

**Fig 4:**
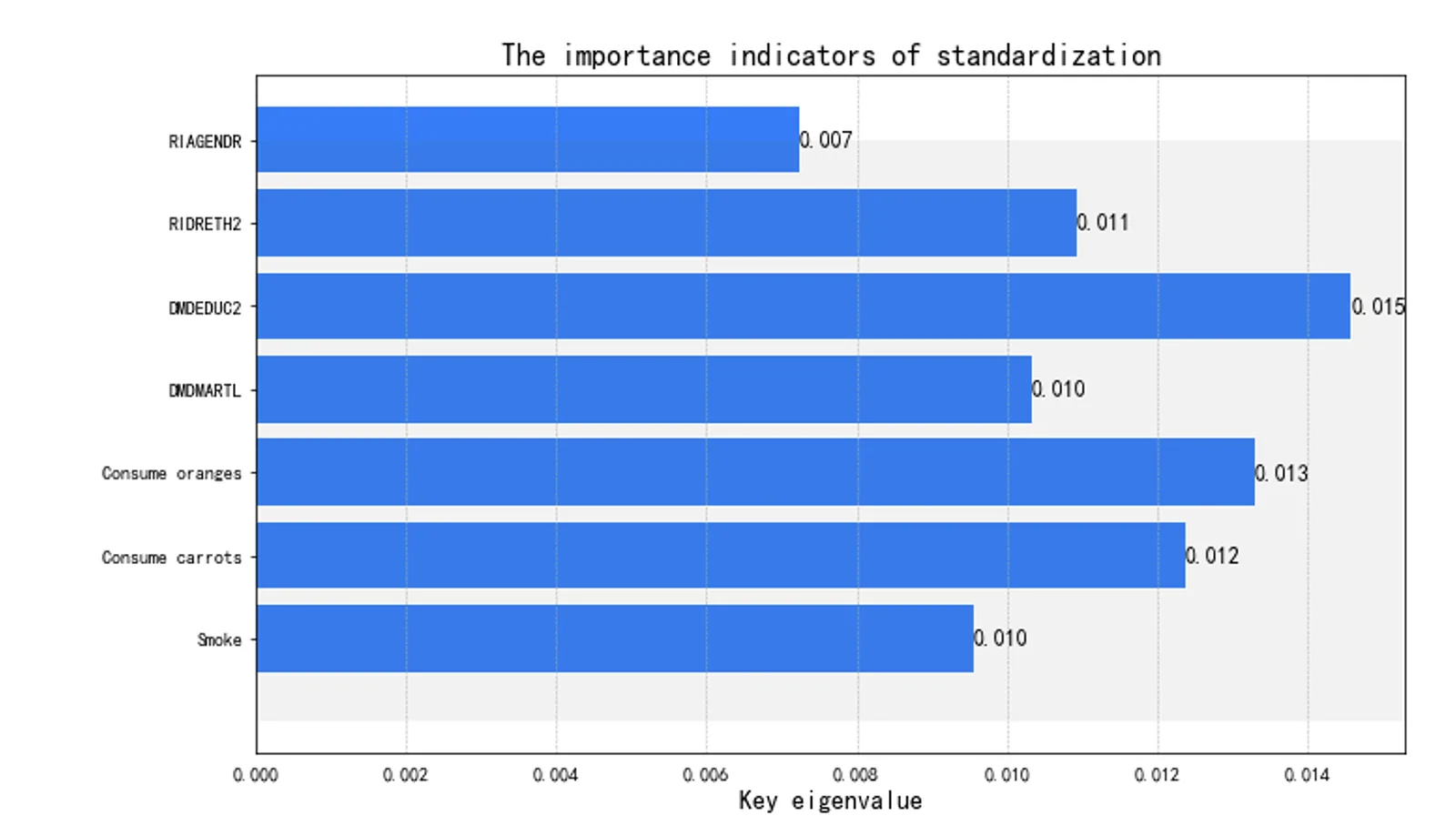
Key characteristic value diagrams of each feature during the standardization process.

### Comparison of Model Prediction Results with Actual Results

The confusion matrix (Fig 5) shows strong model performance with category weights applied. Weighted precision was 0.9658, weighted recall was 0.9710, and weighted F1 score was 0.9682, all above 0.96.

**Fig 5:**
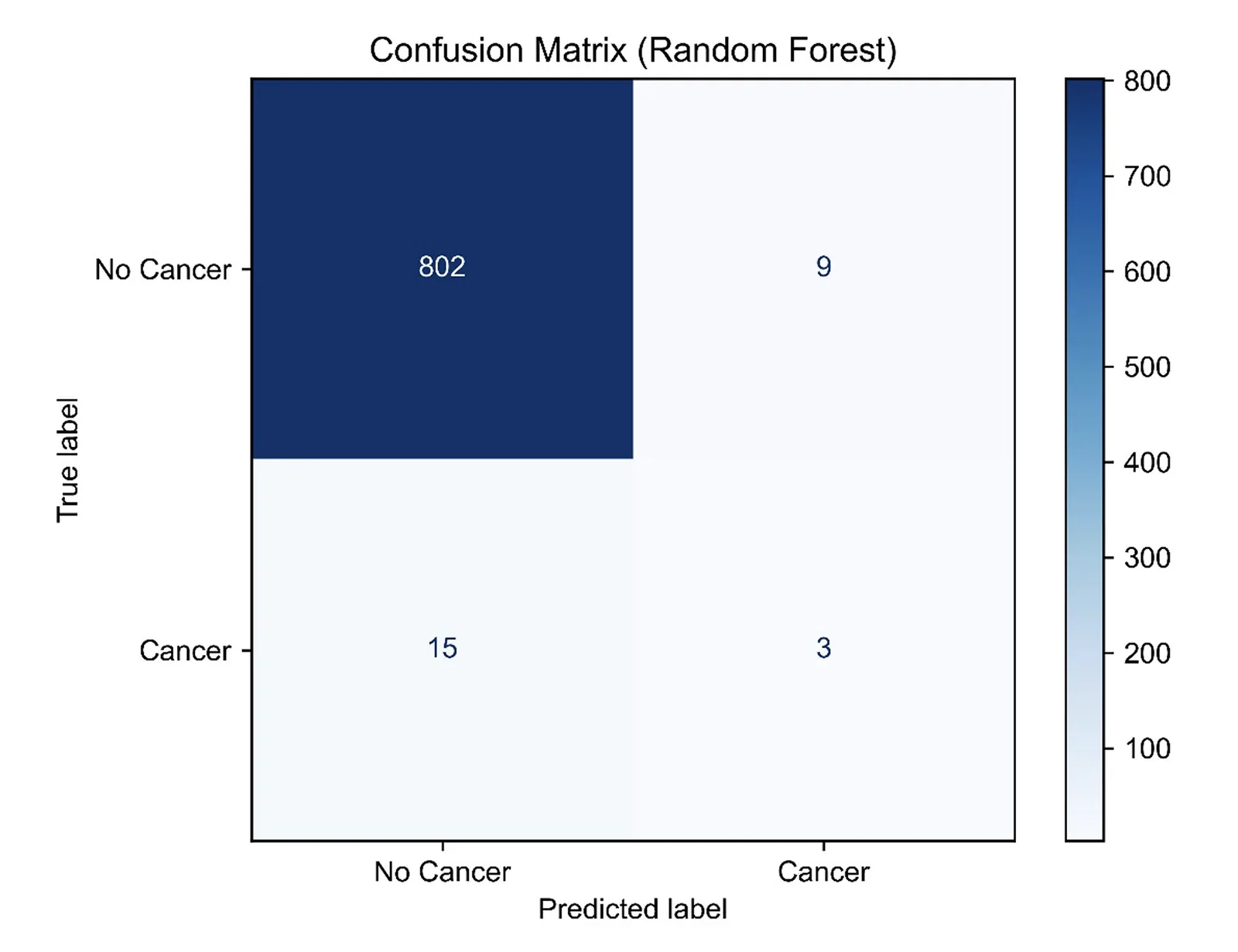
Confusion matrix presenting the performance of the model in predicting oral cancer. The x-axis represents the predicted label, which can be “No Cancer” or “Cancer”. The y-axis represents the true label, also “No Cancer” or “Cancer”. The numbers in the matrix indicate the sample counts. The top-left cell (802) shows the number of samples correctly predicted as no cancer, and the top-right cell (9) shows the number incorrectly predicted as cancer. The bottom-left cell (15) shows the number incorrectly predicted as no cancer, and the bottom-right cell (3) shows the number correctly predicted as cancer. The matrix shows the model’s performance in predicting oral cancer, with color intensity reflecting the number of samples, where darker colors indicate more samples and lighter colors fewer.

### Receiver Operating Characteristic Curve and Performance Evaluation Graph of the Model

The ROC curve (Fig 6) demonstrates good model performance with an Area Under the Curve (AUC) of 0.89, indicating effective distinction between oral cancer patients and non-cancer individuals.

**Fig 6:**
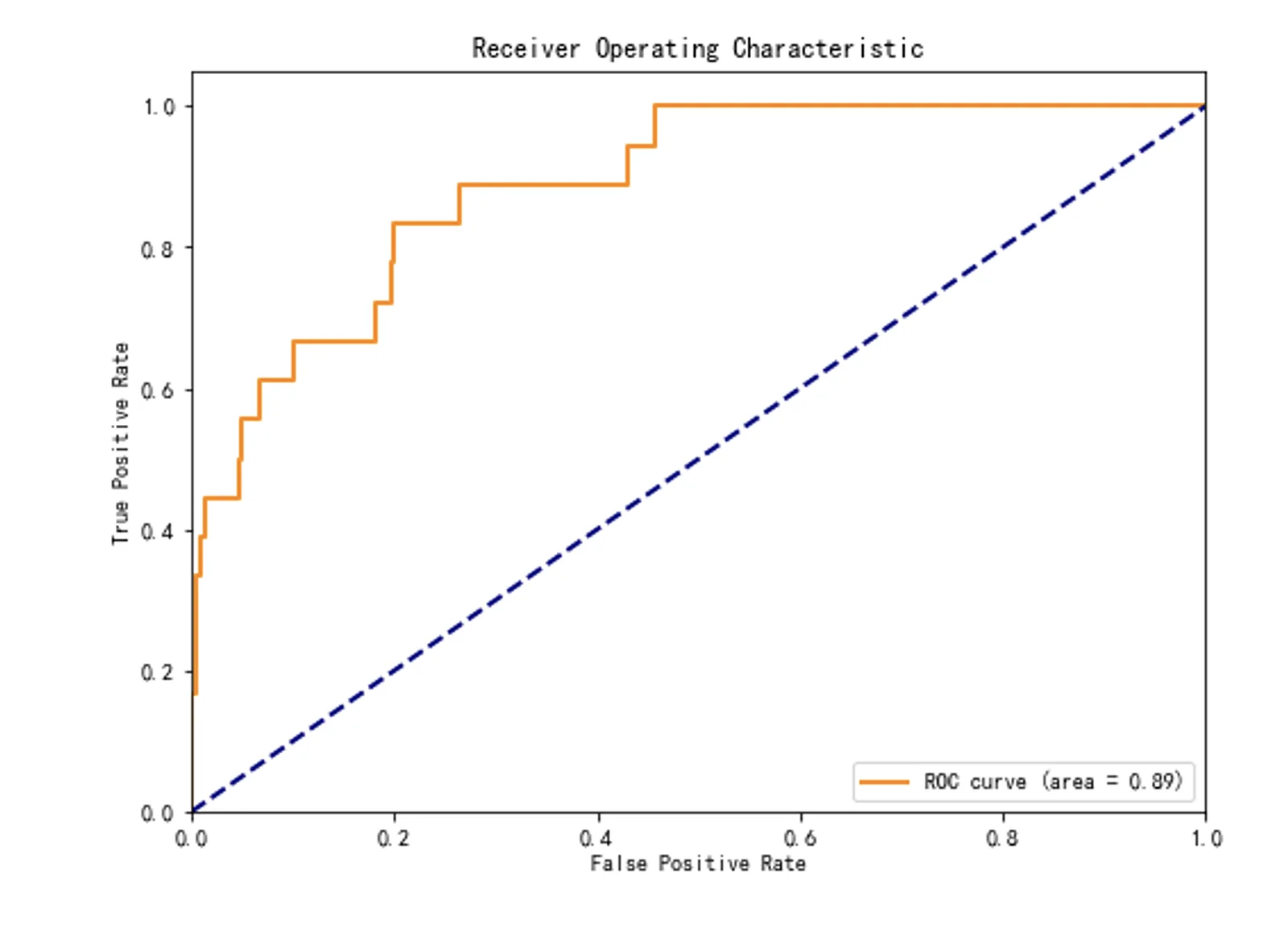
Performance of the predictive model for oral cancer using the ROC (Receiver Operating Characteristic) curve. The curve plots the true positive rate against the false positive rate at various threshold settings. The area under the curve (AUC) is 0.89, indicating excellent discriminatory ability of the model in distinguishing between individuals with and without oral cancer. The closer the curve is to the top-left corner, the better the model’s predictive accuracy. The dashed line represents the ROC curve of a purely random classifier, while the orange line represents the performance of our predictive model. The results suggest that the model has a high capacity to predict oral cancer risk effectively.

## DISCUSSION

Through multi-factor analysis, this study found that socioeconomic status, lifestyle, and dietary conditions containing curcumin were associated with oral cancer. The proportion of those not consuming foods containing curcumin was higher in the oral cancer group, suggesting that foods containing curcumin may have a potential role in preventing oral cancer. Correlation analysis indicated that educational level, and poverty-income ratio were negatively correlated with oral cancer, and dietary conditions including oranges and carrots (foods containing curcumin) were negatively correlated with oral cancer. Logistic regression analysis revealed that occasional and frequent consumption of oranges were associated with reduced odds of oral cancer. Trend tests showed that as the frequency of orange and carrot intake increased, the proportion of oral cancer cases decreased statistically significantly, further confirming the negative correlation between frequency of intake and risk of disease. The 3D bubble chart visually demonstrated the association between the frequency of intake of oranges/carrots and oral cancer. High frequency intake of oranges or carrots was associated with a lower risk of oral cancer, and high frequency intake of both was also associated with a lower risk. Additionally, SHAP-value analysis highlighted the crucial role of a diet containing curcumin in the model, the confusion matrix showed that the model’s predictive ability for the minority class improved statistically significantly, the weighted precision, recall rate, and F1 score were all above 0.96, and the ROC curve verified that the model’s AUC reached 0.89, indicating its good performance in predicting oral cancer.

From the perspective of socioeconomic factors, the groups with lower educational levels and lower income are more likely to be present in the oral cancer group. This may be related to the fact that these groups have insufficient knowledge about oral health, poor oral hygiene habits, and neglect of the early symptoms of oral cancer.^8,13,23^ In terms of lifestyle, smoking can cause damage to the oral mucosa, promote cancer development, and statistically significantly increase the incidence of oral cancer. Alcohol consumption can also influence the oral mucosa and long-term and excessive drinking increases the risk of developing oral cancer.^3,16,28^ Furthermore, smoking and drinking alcohol often occur simultaneously, and their combined effect further increases the risk of oral cancer.^2^

Curcumin, as a natural compound, has significant biological activity. It can regulate various signaling pathways within cells, thereby influencing cell proliferation, differentiation, and apoptosis processes. The antioxidant properties of curcumin enable it to neutralize free radicals, reduce oxidative stress damage to cells, and thereby lower the risk of cancer occurrence.^18,28^ Furthermore, curcumin can also enhance the body’s ability to monitor and eliminate tumor cells by regulating the immune system.^4^ In daily diet, foods such as oranges and carrots are important sources of curcumin. These foods not only contain curcumin but also various other vitamins and minerals, which work together to maintain the health of the body. This study found that people who frequently consume these foods have a lower risk of developing oral cancer. This may be related to the long-term accumulation of curcumin in the body, which helps to continuously inhibit potential carcinogenic factors.^25^

The results of the trend test further indicate that as the frequency of orange and carrot intake increases, the incidence rate of oral cancer shows a statistically significant downward trend (with p-values all less than 0.01), highlighting a statistically significant negative correlation between intake frequency and the prevalence of oral cancer. This is consistent with previous research results, as studies have found that curcumin can inhibit the proliferation and invasion of oral cancer cells, induce cell apoptosis, and thereby reduce the risk of oral cancer.^26^ Furthermore, the interaction analysis shown in the 3D bubble chart also reveals that frequent consumption of oranges or carrots is associated with a lower risk of oral cancer. Additionally, frequent consumption of both oranges and carrots is linked to a lower risk, while low-frequency consumption of both is associated with a higher risk. Relevant studies suggest that the metabolism of curcumin in the body may affect its anti-cancer effect. It is absorbed during digestion and distributed throughout the body through the bloodstream. In tissues such as the oral mucosa, curcumin can interact with local cells and directly exert its biological effects. This direct action may play a crucial role in preventing the occurrence of oral cancer.^14^ The synergistic effect of curcumin in the diet and other nutrients may also have an impact on the prevention of oral cancer. For instance, antioxidants such as vitamin C and E can enhance the antioxidant effect of curcumin, while dietary fiber helps maintain the health of the digestive system and promotes the absorption and utilization of curcumin. This dietary synergy may further reduce the risk of developing oral cancer.^13,20,27^

This study also found that people with higher educational levels and higher incomes tend to have better dietary habits, pay more attention to oral hygiene, undergo regular oral examinations, and be able to detect oral problems at an early stage and seek medical treatment promptly, thereby reducing the risk of developing oral cancer.^6^

Although the NHANES database does not provide absolute quantitative data on curcumin content (mg/day), we attempted to estimate a potential preventive dose based on the consumption frequency categories. According to previous literature, one serving of curry or a turmeric-containing dish provides approximately 50–100 mg of curcumin, and a typical serving of carrots or oranges contains trace amounts of curcuminoids (approximately 1–5 mg per serving). Based on the “Frequently eat” category (≥3 times per week) in our study, the estimated weekly curcumin intake would be approximately 150–300 mg, corresponding to a daily intake of roughly 20–40 mg. This estimated range is consistent with previous studies suggesting that daily curcumin intake of 30–50 mg may exert chemopreventive effects. However, due to the well-known low bioavailability of curcumin and the lack of precise food composition data in NHANES, this estimate should be interpreted with caution. Future pharmacokinetic and dose-response studies are warranted to determine the optimal preventive dose.

This study also has certain limitations. Firstly, the research is based on data from a single year, which may lead to selection bias; secondly, the assessment of dietary habits relies on self-reporting, which may be affected by recall bias; finally, the potential mechanism of curcumin in relation to oral cancer was not deeply explored. Despite these limitations, the results of this study still provide new ideas and a scientific basis for the prevention and risk assessment of oral cancer, and be important for public health. Future research can expand the sample size, extend the period, and more comprehensively investigate the internal mechanism and clinical application prospects of foods containing curcumin in terms of oral cancer prevention, and further improve the cognitive framework of knowledge in this field.

Our findings should be critically interpreted in the context of the existing literature. While our results support the protective association between curcumin-containing foods and oral cancer, some clinical trials have reported limited chemopreventive effects of curcumin due to its poor bioavailability. The discrepancy may be explained by differences in study design: our observational study reflects long-term dietary exposure, whereas short-term supplementation trials may not capture the cumulative effects of curcumin. Additionally, curcumin in dietary sources is often consumed with fats and piperine (e.g., in curry), which enhance its absorption, whereas purified curcumin supplements may have lower bioavailability.

From a clinical perspective, our findings suggest that dietary counseling on regular consumption of curcumin-containing foods could be integrated into oral cancer prevention strategies, particularly for high-risk populations (e.g., smokers and heavy drinkers). Based on our estimated threshold (≥3 times per week of oranges or carrots, corresponding to ~20–40 mg curcumin daily), clinicians may recommend patients to incorporate turmeric, oranges, and carrots into their daily diet as a low-cost, low-risk adjunctive preventive measure.

Future research should prioritize: (1) prospective cohort studies with repeated dietary assessments and quantitative measurement of curcumin intake (mg/day); (2) randomized controlled trials evaluating the efficacy of curcumin supplementation in high-risk populations; (3) mechanistic studies investigating the interaction between curcumin and oral microbiome in carcinogenesis; and (4) pharmacodynamic studies to determine the optimal dosing and formulation of curcumin for oral cancer prevention.

## CONCLUSION

The consumption of foods containing curcumin in the daily diet may reduce the risk of oral cancer through multiple biological mechanisms. This finding provides new ideas for preventing oral cancer through dietary intervention and emphasizes the importance of a balanced diet in cancer prevention. However, this was a cross-sectional study and cannot directly infer causality. Moreover, due to database limitations, the intake content of curcumin and other potential confounding factors were not included. Based on the limitations of the NHANES database sample, future studies still need to combine comprehensive databases or conduct prospective cohort studies to further verify.

## References

[ref4] Bose S, Panda AK, Mukherjee S, Sa G (2015). Curcumin and tumor immune-editing: resurrecting the immune system. Cell Div.

[ref6] Duijster D, Oude Groeniger J, van der Heijden G, van Lenthe FJ (2018). Material, behavioural, cultural and psychosocial factors in the explanation of socioeconomic inequalities in oral health. Eur J Public Health.

[ref7] Eke PI, Wei L, Thornton-Evans G, Borgnakke WS (2025). Scoring algorithm for predicting periodontitis in dentate adults using self-report measures – National Health and Nutrition Examination Survey 2009-2012. Periodontol 2000.

[ref8] Eke PI, Wei L, Thornton-Evans G, Greenlund K, Borgnakke WS (2025). Missing teeth and all-cause mortality in US adults. Periodontol 2000.

[ref9] Gassib N, Issa H, Loubaki L, Behaz S, Almutairi MH, Rouabhia M (2024). Cellular mechanisms mediating the anti-cancer effects of carnosol on gingiva carcinoma. Sci Rep.

[ref10] Hecht SS, Hatsukami DK (2022). Smokeless tobacco and cigarette smoking: chemical mechanisms and cancer prevention. Nat Rev Cancer.

[ref11] Isola G, Polizzi A, Serra S, Boato M, Sculean A (2025). Relationship between periodontitis and systemic diseases: A bibliometric and visual study. Periodontol 2000.

[ref12] Jia Z, Chen K (2024). Association between the Composite Dietary Antioxidant Index and periodontitis in US adults: a cross-sectional analysis. Quintessence Int.

[ref13] Joshi V, Joshi NK, Bajaj K (2022). Tobacco use pattern, dependence, oral cancer awareness and health education needs among urban slum dwellers of Jodhpur City. Int J Prev Med.

[ref14] Krishna Rao SV, Mejia G, Roberts-Thomson K, Logan R (2013). Epidemiology of oral cancer in Asia in the past decade – an update (2000-2012). Asian Pac J Cancer Prev.

[ref15] Li N, Chen X, Liao J, Yang G, Wang S, Josephson Y (2002). Inhibition of 7,12-dimethylbenza.anthracene (DMBA)-induced oral carcinogenesis in hamsters by tea and curcumin. Carcinogenesis.

[ref16] Merletti F, Boffetta P, Ciccone G, Mashberg A, Terracini B (1989). Role of tobacco and alcoholic beverages in the etiology of cancer of the oral cavity/oropharynx in Torino, Italy. Cancer Res.

[ref17] Patel SS, Acharya A, Ray RS, Agrawal R, Raghuwanshi R, Jain P (2020). Cellular and molecular mechanisms of curcumin in prevention and treatment of disease. Crit Rev Food Sci Nutr.

[ref18] Peng Y, Peng Y (2026). Association between stress hyperglycemia ratio and periodontitis among diabetes: evidence from National Health and Nutrition Examination Survey 2009-2014. Quintessence Int.

[ref19] Radaic A, Kamarajan P, Cho A, Wang S, Hung G-C, Najarzadegan F (2024). Biological biomarkers of oral cancer. Periodontol 2000.

[ref20] Rai B, Kaur J, Jacobs R, Singh J (2010). Possible action mechanism for curcumin in pre-cancerous lesions based on serum and salivary markers of oxidative stress. J Oral Sci.

[ref21] Rajasekaran SA (2011). Therapeutic potential of curcumin in gastrointestinal diseases. World J Gastrointest Pathophysiol.

[ref22] Reis SR, do Espírito Santo AR, Andrade MG, Sadigursky M (2006). Cytologic alterations in the oral mucosa after chronic exposure to ethanol. Braz Oral Res.

[ref23] Smith DK, Murphy BA (2019). Lower levels of education and household income mediate lower dental care utilization among survivors of early life cancers. Prev Med Rep.

[ref24] Soghli N, Khormali A, Mahboubi D, Peng A, Miguez PA (2025). Recent advancements in artificial intelligence-powered cancer prediction from oral microbiome. Periodontol 2000.

[ref25] Taghavi N, Yazdi I (2007). Type of food and risk of oral cancer. Arch Iran Med.

[ref26] Zhang H, Kim E, Yi J (2021). [6]-gingerol suppresses oral cancer cell growth by inducing the activation of AMPK and suppressing the AKT/mTOR signaling pathway. In Vivo.

[ref28] Znaor A, Brennan P, Gajalakshmi V, Mathew A, Shanta V, Varghese C (2003). Independent and combined effects of tobacco smoking, chewing and alcohol drinking on the risk of oral, pharyngeal and esophageal cancers in Indian men. Int J Cancer.

